# Patient perspectives on 3-monthly paliperidone long-acting injections (PP3M): real-world questionnaire data from South London community mental health teams

**DOI:** 10.1192/j.eurpsy.2026.10538

**Published:** 2026-07-31

**Authors:** I. Clark, S. Gee, D. Taylor

**Affiliations:** 1 Institute of Pharmaceutical Science, King’s College London; 2 Pharmacy, South London and Maudsley NHS Foundation Trust, London, United Kingdom

## Abstract

**Introduction:**

PP3M has been proven to reduce relapse risk, hospital admissions and days spent in hospital in patients with schizophrenia, but there is little real-world evidence on patient acceptability and experience.

**Objectives:**

To assess patients’ experience, preferences, tolerability with PP3M compared to the monthly formulation (PP1M).

**Methods:**

We distributed a 10-item questionnaire to adults on PP3M in community teams in four South London boroughs between July 2024 and March 2025. The domains covered were helpfulness, side effects, injection pain, service contact, and preferred dosing intervals. Descriptive analyses (percentages, non-parametric tests) were conducted via SPSS v29.

**Results:**

68 of 172 eligible patients responded (39.5%). 75% rated PP3M as “very” or “extremely” helpful; 84% preferred PP3M over PP1M, mostly citing convenience (62.9%) followed by medication efficacy (22.9%). Injection pain was mild or absent in 65.1%. When asked to describe their experience of adverse effects, 58.7% reported none, 25.4% reported experiencing some but they were not troubling, 11.1% had somewhat troubling and 4.8% reported unbearable adverse effects. No *new* adverse effects with PP3M were reported by 80.6%. Mental health service contact remained unchanged for 55.6%, while 31.7% reported more contact and 12.7% reported less contact. For preferred dosing interval, 64.5% preferred 3-monthly dosing; 33.9% would prefer 6-monthly, 1.6% would prefer 1-monthly dosing and 0% preferred one- or two-weekly interval.

**Conclusions:**

Patients on PP3M report high acceptability, greater tolerability and strong preference for PP3M. Stable patients on PP1M should be offered the option to change to 3-monthly or 6-monthly paliperidone.

**Disclosure of Interest:**

I. Clark Speakers bureau of: honoraria from Jannsen for presentation delivery, S. Gee: None Declared, D. Taylor Shareholder of: Myogenes, Grant / Research support from: Janssen, Recordato, Speakers bureau of: Janssen, Recordati, Sunovion, Otsuka.

